# Quinolone Susceptibility and Detection of *qnr* and *aac*(6’)-*Ib-cr* Genes in Community Isolates of *Klebsiella pneumoniae*

**DOI:** 10.5812/jjm.11136

**Published:** 2014-07-01

**Authors:** Seyed Mohsen Seyedpour, Fereshteh Eftekhar

**Affiliations:** 1 Department of Microbiology, Faculty of Biological Sciences, Shahid Beheshti University, G.C., Tehran, IR Iran

**Keywords:** *Klebsiella Pneumonia*, Quinolone Resistance, PMQR, qnr, aac (6’)-Ib-cr qnr Genes

## Abstract

**Background:**

Plasmid-mediated quinolone resistance genes (PMQR) have been shown to play not only an important role in quinolone resistance, but also resistance to other antibiotics, particularly β-lactams and aminoglycosides. These genes are mainly associated with clinical isolates of *Enterobacteriaceae.* However, detection of PMQR genes in the community isolates can increase the dissemination rate of resistance determinants among bacteria.

**Objectives:**

This study aimed to investigate quinolone resistance and distribution of *qnr* and *aac* (6’)-*Ib-cr* genes among the community isolates of *Klebsiella pneumoniae*.

**Materials and Methods:**

Fifty-two *K. pneumoniae* isolates were collected from the Central Laboratory in Karaj between July 2010 and January 2011. Antibacterial susceptibility was determined by the disc diffusion method. Quinolone and/or cephalosporin-resistant isolates were screened for the presence of *qnr*A, *qnr*B, *qnr*S and *aac* (6’)-*Ib-cr* genes by polymerase chain reaction (PCR).

**Results:**

Of the 52 *K. pneumoniae* isolates, 23 were resistant to cephalosporins and/or quinolones. Overall, 7 out of the 23 resistant isolates harbored *qnr* and/or *aac* (6’)-*Ib-cr* genes (30.4%). Among these, 5 isolates were resistant to both classes of antibiotics of which; 3 carried the *aac* (6’)-*Ib-cr* gene, one had the *qnr*S, and one harbored both *aac* (6’)-*Ib-cr* and *qnr*B genes. None of the isolates contained *qnr*A. Two isolates were sensitive to quinolones and resistant to cephalosporins of which; one had *qnr*S and the other carried the *aac* (6’)-*Ib-cr* gene.

**Conclusions:**

Our study showed that 30.4% of the quinolone and/or cephalosporin resistant community isolates of* K. pneumoniae* carried PMQR genes. These results confirm that community isolates can be an important source for spreading antibiotic resistance determinants among Gram negative pathogens. This is the first report from Iran on detection of PMQR in the community isolates of *K. pneumoniae*.

## 1. Background

*Klebsiella pneumoniae* is an opportunistic pathogen responsible for up to 10% of all nosocomial infections (1, 2). These infections are often treated with extended-spectrum cephalosporins, fluoroquinolones and carbapenems. However, resistance mechanisms such as production of β-lactamases, plasmid-mediated quinolone resistance (PMQR) and carbapanemases by the organisms have created serious therapeutic problems (3-5).

 PMQR determinants comprise; *Qnr*A, *Qnr*B, *Qnr*S, *Qnr*C and *Qnr*D proteins which protect DNA gyrase and topoisomerase IV from inhibition by quinolones; the aminoglycoside acetylteransferase variant, *aac (6’)–Ib–cr *capable of acetylating and subsequently reducing the activity of norfloxacin and ciprofloxacin; and finally, the recently described flouroquinolone specific efflux pump protein, qepA (5). Although PMQR determinants confer low level of quinolone resistance on their own, they have been shown to facilitate the acquisition of high level resistance among initially susceptible strains (6, 7).

 PMQR determinants have been mostly identified in clinical isolates of *Enterobacteriaceae*, including *K. pneumoniae*, and have been shown to play not only an important role in quinolone resistance, but also resistance to other antibiotics, particularly β-lactams and aminoglycosides (8, 9). In fact, a number of studies have shown the presence of *qnr* genes along with various lactamases determinants on the same plasmids (10-14). Presence of PMQR genes in the community isolates of *K. pneumoniae* has also been shown, which provides a wider reservoir for the spread of these organisms (15).

## 2. Objectives

We studied the presence of *qnr*A, *qnr*B, *qnr*S and *aac* (6’)–*ib–cr* determinants among the cephalosporin and/or quinolone resistant community isolates of *K. pneumoniae*.

## 3. Materials and Methods

### 3.1. Bacteria

Fifty-two *K. pneumoniae* isolates were collected from the Central laboratory in Karaj between July 2010 and January 2011 of which, 80.8% were from urine and 19.2% from stool samples. All isolates were identified by conventional biochemical and microbiological tests and were maintained in brain heart infusion broth (Oxoid, UK) containing 10% dimethyl sulfoxide (v/v) at -20ºC until use.

### 3.2. Antibacterial Susceptibility

Susceptibility to antibiotics was determined by the disc diffusion method using the CLSI recommendations and the following antibiotics (Himedia, India): amoxiclav (AMC, 20+10 µg), aztreonam (ATM, 30 µg), cefepime (CPM, 30 µg), cefotaxime (CTX, 30 µg), ceftazidime (CAZ, 30 µg), imipenem (IPM, 10 µg), ciprofloxacin (CP, 30 µg), levofloxacin (LOM, 5 µg), norfloxacin (NOR, 10 µg), ofloxacin (OFX, 5 µg) and nalidixic acid (NA, 30 µg). *K. pneumoniae* ATCC 10031 was used as the quality control for antimicrobial susceptibility tests.

### 3.3. DNA Extraction and PCR Amplification

DNA extraction was performed using an improved phenol/chloroform method where the lysis step was eliminated, and the cells were lysed directly by phenol (16). Presence of *qnr*A, *qnr*B, *qnr*S, and *aac* (6’)-*Ib-cr* genes was detected by PCR using the primers shown in Table 1 (12, 17). The reaction mixture (25 µl) contained 1.5 µl DNA template, 1.5 mM MgCl2, 0.25 mM of dNTP mix (Cinnagen, Iran), 1 unit of DFS-Taq DNA polymerase (Bioron, Germany), and 20 pmol of each primer (Faza Biothec, Iran). Amplifications were performed in a thermal cycler (Bioer TC25/H, Bioer Technolongy, China) using the following program: initial denaturation at 94ºC for 5 min followed by 30 cycles of 1 min at 94 ºC, 1 min at annealing temperature (57ºC for *qnr*A, *qnr*B and *qnr*S, 54ºC for *aac* (6’)-*Ib-cr*), 1 min at 72ºC and a final extension period of 10 min at 72 C. The amplified PCR products were resolved by electrophoresis in 1.5% agarose gel and visualized after staining with ethidium bromide (Merck, Germany).

**Table 1.** Primers Used For Detection of *qnr* and *aac* (6’)-*Ib-cr* Genes

Gene	Primer Type	Primer Sequence	PCR Product Size	Reference
***qnrA***	Forward	TTCTCACGCCAGGATTTGAG	571 bp	(17)
	Reverse	TGCCAGGCACAGATCTTGAC		
***qnrB***	Forward	TGGCGAAAAAATTG*AAC*AGAA	594 bp	(17)
	Reverse	GAGC*AAC*GATCGCCTGGTAG		
***qnrS***	Forward	GACGTGCT*AAC*TTGCGTGAT	388 bp	(17)
	Reverse	*AAC*ACCTCGACTTAAGTCTGA		
***aac(6’)-Ib-cr***	Forward	TTGCGATGCTCTATGAGTGGCTA	482 bp	(12)
	Reverse	CTCGAATGCCTGGCGTGTTT		

## 4. Results

### 4.1. Antibacterial Susceptibility

**Figure 1.**
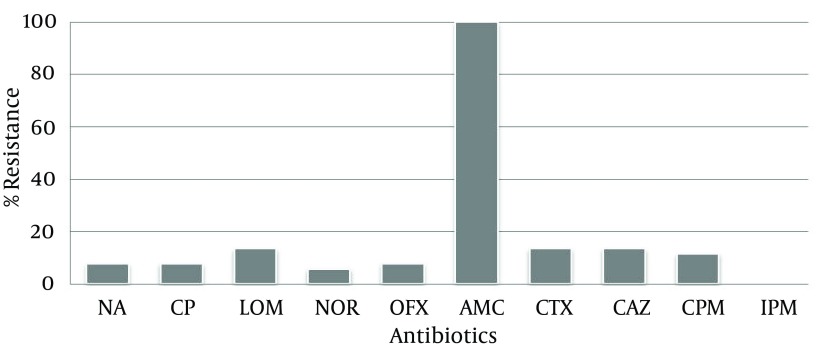
Antibiotic Resistance Profile of 52 *K. pneumoniae* Isolates Collected From Outpatients. NA: nalidixic acid, CP: ciprofloxacin, LOM: levofloxacin, NOR: norfloxacin, IPM: Imipenem, OFX: ofloxacin, AMC: amoxiclav, CTX: cefotaxime, CAZ: ceftazidime, CPM: cefepime.

The antibiotic susceptibility results of the 52 *K. pneumoniae* isolates are shown in Figure 1. All isolates were resistant to amoxilcav and susceptible to imipenem. Resistance rates to the other antibiotics were 13.5% to ceftazidime, cefotaxime and levofloxacin; 11.5% to cefepime; 7.7% to ciprofloxacin, nalidixic acid, and ofloxacin; and 5.8% to norfloxacin (Figure 1). Twenty-three isolates were chosen for PCR studies based on their resistance to quinolones and/or cephalosporins.

### 4.2. Detection of qnr and aac (6’)-Ib-cr Determinants

**Figure 2.**
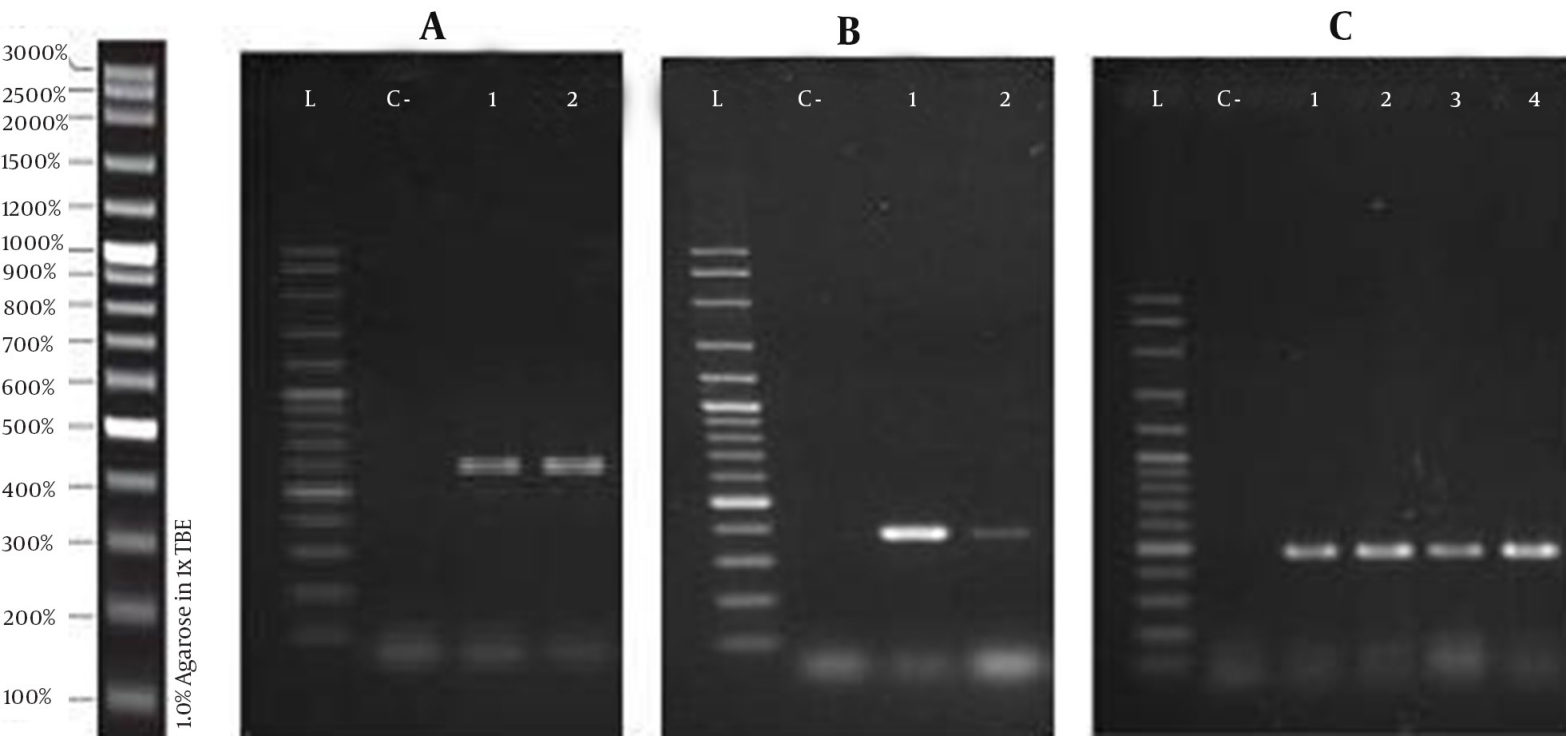
PCR Amplification Products of *qnr* and *aac* (6’)-*Ib-cr* Genes A, *qnrB*; B, *qnrS* and C, *aac*(6’)-*Ib-cr* genes in 23 community isolates of *K. pneumoniae*. L, 1 Kbp ladder; C-, negative control.

Figure 2 shows the PCR amplification products of *qnr* and *aac* (6’)-*Ib-cr* genes among the 23 selected isolates. Overall, 7 out of the 23 selected isolates harbored *qnr* and/or *aac* (6’)-*Ib-cr* genes (30.4%), 6 of which were urinary isolates and 1, a stool isolate. None of the isolates harbored *qnr*A. Five isolates were resistant to all test quinolones and cephalosporins, of which, 3 carried *aac* (6’)-*Ib-cr*, one had *qnr*S, and one carried both *aac* (6’)-*Ib-cr* and *qnr*B genes. *Qnr*B was detected in 2 isolates both of which were quinolone and cephalosporin resistant. Two isolates harbored the *qnr*S gene; one of which was resistant to quinolones and cephalosporins and the other was quinolone susceptible, cephalosporin resistant. Finally, one quinolone susceptible, cephalosporin resistant isolate carried the *aac* (6’)-*Ib-cr* gene.

## 5. Discussion

Presence of *qnr* and *aac* (6’)-*Ib-cr* genes in clinical isolates of *E. coli* and *K. pneumoniae* has been reported worldwide (4-9). A large number of studies have also shown the presence of *qnr* genes along with resistance to various β-lactamases, including the AmpC and extended-spectrum β-lactamases (10, 11, 14, 18, 19). However, studies on the presence of PMQR genes in the community isolates are far fewer. In the present study, majority of the community isolates of *K. pneumoniae* were susceptible to all test antibiotics except for amoxiclav. However, despite the low rate of antibiotic resistance, 13.5% of all test isolates and 30.4% of the quinolone and/or cephalosporin resistant isolates carried *qnr* and/or *aac* (6’)-*Ib-cr* genes.

 In a study conducted on *Escherichia coli* in Italy, the rate of *qnr* gene carriage was 27.8% of which; *aac* (6')-*Ib-cr* was detected in 11% of the community isolates (20). In another study conducted in northern Italy between 2004 and 2006, the *aac* (6')-*Ib-cr* gene was found in 3.9% of the community isolates of the uropathogenic *E. coli* (21). PMQR genes were also reported in commensal isolates of *Enterobacteriaceae* from Vietnam, including 45 *K. pneumoniae* isolates of which 35.5% carried the *qnr*S and *aac* (6')-*Ib-cr* genes (22). More recently, a study from Morocco showed that among 34 community isolates of *K. pneumoniae*, 41% harbored plasmid-mediated *qnr* genes, including *qnr*A, *qnr*B and *qnr*S, and 76.4% carried the *aac* (6')-*Ib-cr* gene (15). Our results were closer to the report from Vietnam but much lower than the Moroccan study. We did not detect the *qnr*A gene among our isolates. Although *qnr*A1 was the first PMQR gene discovered, several studies have indicated that *qnr*S, *qnr*B and *aac* (6’)-*Ib-cr*) are more commonly found among* Enterobacteriaceae* (5, 6, 23).

 Consistent with the previous studies, we also showed the presence of *aac* (6')-*Ib-cr*, *qnr*B, and *qnr*S genes but not *qnr*A among our community isolates. We believe that this is the first report on the presence of PMQR genes in *K. pneumoniae* isolates collected from outpatients in Iran. There is one other study from Iran where the prevalence of PMQR genes (*qnr*A and *qnr*B but not *qnr*S) was detected in *E. coli* (13). Since quinolone resistant genes are plasmid-mediated, dissemination of these antibiotic resistance determinants could easily occur between opportunistic Gram-negative pathogens, which can be problematic and further limit treatment of these infections.
